# Incidence and locations of deep venous thrombosis of the lower extremity following surgeries of tibial plateau fractures: a prospective cohort study

**DOI:** 10.1186/s13018-020-02136-0

**Published:** 2020-12-14

**Authors:** Junyong Li, Yanbin Zhu, Wei Chen, Kuo Zhao, Junzhe Zhang, Hongyu Meng, Zhucheng Jin, Dandan Ye, Yingze Zhang

**Affiliations:** 1 Department of Orthopaedic Surgery, The 3rd Hospital of Hebei Medical University, Shijiazhuang, 050051 Hebei PR China; 2 Key Laboratory of Biomechanics of Hebei Province, Shijiazhuang, 050051 Hebei PR China; 3 Orthopaedic Institution of Hebei Province, Shijiazhuang, 050051 Hebei PR China; 4 The Second Hospital of Shijiazhuang City, Shijiazhuang, 050051 Hebei PR China; 5 NHC Key Laboratory of Intelligent Orthopeadic Equipment, Hebei 050051 Shijiazhuang, People’s Republic of China

**Keywords:** Deep venous thrombosis, Surgical intervention, Tibial plateau fractures, Incidence, Risk factors

## Abstract

**Objective:**

To investigate the incidence of deep venous thrombosis (DVT) of the lower extremities following surgeries of tibial plateau fractures.

**Methods:**

Retrospective analysis of the prospectively collected data on patients undergoing surgeries of tibial plateau fractures between October 2014 and December 2018 was conducted. Duplex ultrasonography (DUS) was used to screen for postoperative DVT of the bilateral lower extremities. Data on demographics, comorbidities, injury, surgery, and laboratory biomarkers at admission were collected. Univariate analyses and multivariate logistic regression analyses were used to identify the independent risk factors associated with DVT.

**Results:**

Among 987 patients included, 46 (4.7%) had postoperative DVT, with incidence rate of 1.0% for proximal and 3.7% for distal DVT. The average interval between operation and DVT was 8.3 days (median, 5.8 days), ranging from 2 to 42 days. DVT involved the injured extremity in 39 (84.8%) patients, both the injured and uninjured extremity in 2 patients (4.3%) and only the uninjured extremity in 5 patients (10.9%). Five risk factors were identified to be associated with postoperative DVT, including age (≥ 41 vs < 41 years) (OR 3.08; 95% CI 1.43–6.61; *p* = 0.004), anesthesia (general vs regional) (OR 2.08; 95% CI 1.12–3.85; *p* = 0.021), hyponatremia (OR 2.21; 95% CI 1.21–4.06; *p* = 0.010), prolonged surgical time (OR 1.04; 95% CI 1.01–1.07; *p* = 0.017) and elevated D-dimer level (OR 2.79; 95% CI 1.34–4.83; *p* = 0.004).

**Conclusion:**

These epidemiologic data may be helpful in individualized assessment, risk stratification, and development of targeted prevention programs.

## Introduction

As is well known, deep venous thrombosis (DVT) is a significant cause of morbidity, pulmonary embolism, and even mortality in all hospitalized patients, especially in the setting of trauma [1, 2]. Tibial plateau fracture, a commonly seen knee injury, represents 1–2% of adult fractures and 32% of peri-knee fractures [3]. The post-injury hypercoagulation state, trauma stress, and systemic inflammatory response are early factors that contributed to the occurrence of DVT. Prolonged duration of extremity elevation and limited mobility, which is needed to allow the soft tissue envelop safe for surgical interventions aggravates the risk of DVT.

Extensive and deep understanding of the related risk factors is critical for prevention of occurrence of DVT, and it is of more clinical significance to distinguish between proximal and distal venous thrombosis, which allows more accurate diagnose and a more aggressive therapy for the proximal DVT. While DVT has been extensively studied in trauma, such as hip fracture, spinal fracture, pelvic and acetabular fracture, and multiple trauma [4–7], there is still lack of epidemiologic data on DVT following tibial plateau fracture. In most cases, the limited sample size in a single institution is powerless in statistical analyses. Besides, the confounding covariables from multi-aspects as trauma stress, patient comorbidities, or injury itself could affect the occurrence of DVT, and their respective role has not been definitely illuminated.

In this study, we used the prospectively collected data to evaluate the epidemiologic characteristics of postoperative DVT in tibial plateau fractures, including the incidence rate, the locations and of DVTs, and the associated risk factors.

## Methods

The data used in this study were extracted from the database of Surgical Site Infection in Orthopaedic Surgery (SSIOS), in which data were prospectively collected on patients undergoing orthopaedic surgeries between October 1, 2014, and December 31, 2018, with the aim to identify surgical site infection. The SSIOS study was approved by the ethics committee of the 3^rd^ Hospital of Hebei Medical University (NO 2014-015-1), and got the informed consent of all the participants.

### Inclusion and exclusion criteria

Patients included in this study must meet the following criteria: age of 18 years or older, undergoing surgery of tibial plateau fracture, and complete data available from medical records. Patients with pathological (metastatic) or old fracture (> 3 weeks from injury), concurrent fractures in other locations, with preoperative DVT during this hospitalization, with history of DVT or other thrombotic events, or with the current use of anticoagulants due to chronic comorbidities were excluded from this study.

In this study, all patients were preoperatively treated with an inflatable tourniquet at the thigh root of the affected limb with an inflated pressure of 280 mmHg, to reduce the study bias caused by the use of inflatable tourniquets.

During hospitalization stay, all patients received basic thromboprophylaxis, consisting of chemical (low molecular weight heparin (LMWH), 2500–4100 IU once daily, subcutaneous injection) and elevation of the injured lower extremity. Postoperative routine ultrasound examination of patients with deep vein thrombosis (DVT), according to guidelines for the treatment of deep vein thrombosis of the lower extremity, the treatment to be taken includes chemical drugs (low molecular heparin (LMWH), 2500–4100 IU twice daily, subcutaneous injection) and postoperative lower extremity elevation.

### Diagnosis of DVT

DVT was diagnosed in accordance with the Guideline for the Diagnosis and Treatment of Deep Vein Thrombosis (3rd edition) proposed by the Chinese Medical Association [8]. During the postoperative period (the period between the first day after the operation and the period of discharge from the hospital), ultrasound examination of lower extremities was performed on the first day after the operation to determine whether deep vein thrombosis had occurred. After that, every 3 days after surgery routine duplex ultrasonography (DUS) scanning were performed in femoral common vein, superficial and deep femoral vein, popliteal vein, posterior and anterior tibial vein, and peroneal vein of bilateral lower extremities. The criteria of positivity were set as noncompressibility, lumen obstruction or filling defect, lack of respiratory variation in above knee segments, and inadequate flow augmentation to calf and foot compression maneuvers [9]. DVT localized in the popliteal vein or proximally was defined as proximal DVT, and those distal to popliteal vein were defined as distal DVT; if both distal and proximal DVT were present in one patient, he was classified in the proximal DVT group [10]. Superficial or intermuscular vein thrombosis (soleal or gastrocnemius vein thrombosis) were excluded, due to their relatively less clinical significance [11, 12].

### Data collection

Data were from 5 aspects: demographics, comorbidities, injury, surgery, and laboratory biomarkers. The demographic data included age, gender, residence, body mass index (BMI), cigarette smoking, and alcohol consumption. The comorbidities included hypertension, diabetes, chronic heart disease, chronic liver disease, history of any surgery, and allergies to any medications, all of which were self-reported by patients. Injury-related data included injury mechanism, open or closed fracture, fracture classification based on Schatzker classification system. The surgery-realted data included preoperative interval since fracture, fracture reduction mode, American Society of Anesthesiologists (ASA) classification, bone graft, surgical duration, day or night surgery, and perioeprative blood transfusion. The BMI (kg/m^2^) was divided using the criteria recommended by the Chinese working group on obesity: normal (18.5–23.9), underweight (< 18.5), overweight (24.0–27.9), and obesity (≥ 28.0) [13]. Low-energy injury was defined as an injury caused by a fall from a standing height, while fall from a height more than 2 m or motor accidents as high-energy injury.

The biomarkers at admission included total protein (TP) level, albumin (ALB) level, globulin (GLOB), ALB/GLOB, alanine transaminase (ALT), aspartate transaminase (AST), alkaline phosphatase (ALP), lactate dehydrogenase (LDH), uric acid (UA), fasting blood glucose (FBG) level, total bilirubin (TBIL), direct bilirubin (DBIL), indirect bilirubin (IBIL), red blood cell (RBC) count, white blood cell (WBC) count, neutrophile (NEUT) count, lymphocyte (LYM) count, monocyte (MON), hemoglobin (HGB) level, hematocrit (HCT), platelet (PLT), red blood cell distribution width (RDW), platelet distribution width (PDW), mean erythrocyte volume (MCV), mean corpuscular hemoglobin (MCH), mean corpuscular hemoglobin concentration (MCHC), total cholesterol (TC) level, triglyceride (TG) level, low-density lipoprotein (LDL-C) level, high-density lipoprotein (HDL-C) level, very low-density lipoprotein (VLDL) level, sodium concentration (Na+), chloride concentration (Cl-), D-dimer level, and osmotic pressure (OSM).

### Statistical analysis

Continuous variables were expressed by mean and standard deviation (SD) and were evaluated by Student’s *t* test or Mann-Whitney *U* test, as appropriate. The categorical data were expressed as number and percentage (%) and were evaluated by chi-squared or Fisher’s exact test, as appropriate.

Giving the association of D-dimer and advancing age in DVT formation, receiver operating characteristic (ROC) was constructed to determine the optimal cutoff value, above which the risk of DVT was significantly increased. The significance of the ROC curve was tested using the area under the curve (AUC) analysis, with *p* < 0.05 as significance level.

Variables tested as approximately significant in the univariate analyses were entered into the multivariate logistics regression model. Stepwise backward elimination method was used to exclude varaible one by one. Variables with *p* < 0.10 were retained in the final model, and the correlation strength is indicated by odd ratio (OR) and 95% confidence interval (95%CI). The significance level was set as *p* < 0.05. Hosmer-lemeshow (H-L) test was used to evaluate the fitting degree of the final model, and *p* > 0.05 represented the acceptable result. SPSS23.0 was used to perform all the tests (IBM, Armonk, New York, USA).

## Results

Among 987 patients included, 603 (61.1%) were males and 384 (38.9%) were females, with 45.2 years in average (Sd, 13.9; range, 18–82; median, 45.0). There were 638 (64.6%) fractures that were caused by high-energy trauma, and 49 (5.0%) were open fractures. Based on Schatzker classification system, 700 (70.9%) were classified as types I–IV, and 287 (29.1%) as types V–VI.

Postoperatively, 46 patients were diagnosed to have DVT, indicating the incidence of 4.7%. No thrombosis was found in anterior tibial vein. At the other 6 veins, there were 78 clots, representing an average of 1.70 (range, 1 to 5) for each patient. DVT involved the injured extremity in 39 patients, both the injured and uninjured extremity in 2 (4.3%) patient and only the uninjured extremity in 5 (10.9%) patients. DVT involved proximal vein in 10 patients and distal vein in 36 patients, with incidence rate of 1.0% and 3.7%, respectively. The details were 1 in femoral common vein, 3 in superficial and 1 in deep femoral vein, 7 in popliteal vein, 29 in posterior tibial vein, and 37 in peroneal vein. The average interval between operation and DVT was 8.3 days (median, 5.8 days), ranging from 2 to 42 days. Compared to those without postoperative DVT, patients with a postoperative DVT had a significantly longer total hospitalization stay (29.8 ± 26.8 vs 18.6 ± 22.2, *p* = 0.001).

**Fig. 1:**
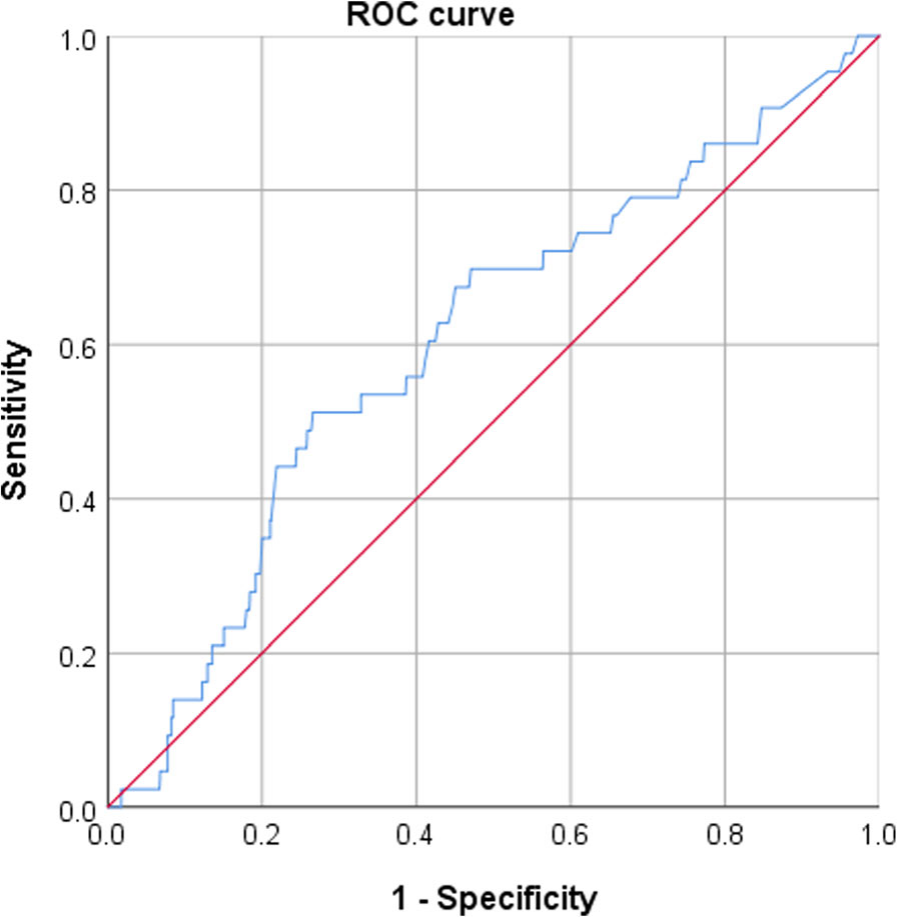
The inflection point of the ROC curve corresponded to D-dimer level above 1.75 mg/L (*p* = 0.021, AUC, 0.604; 95%CI, 0.517 to 0.692; sensitivity = 0.860, specificity of 0.174)

**Fig. 2:**
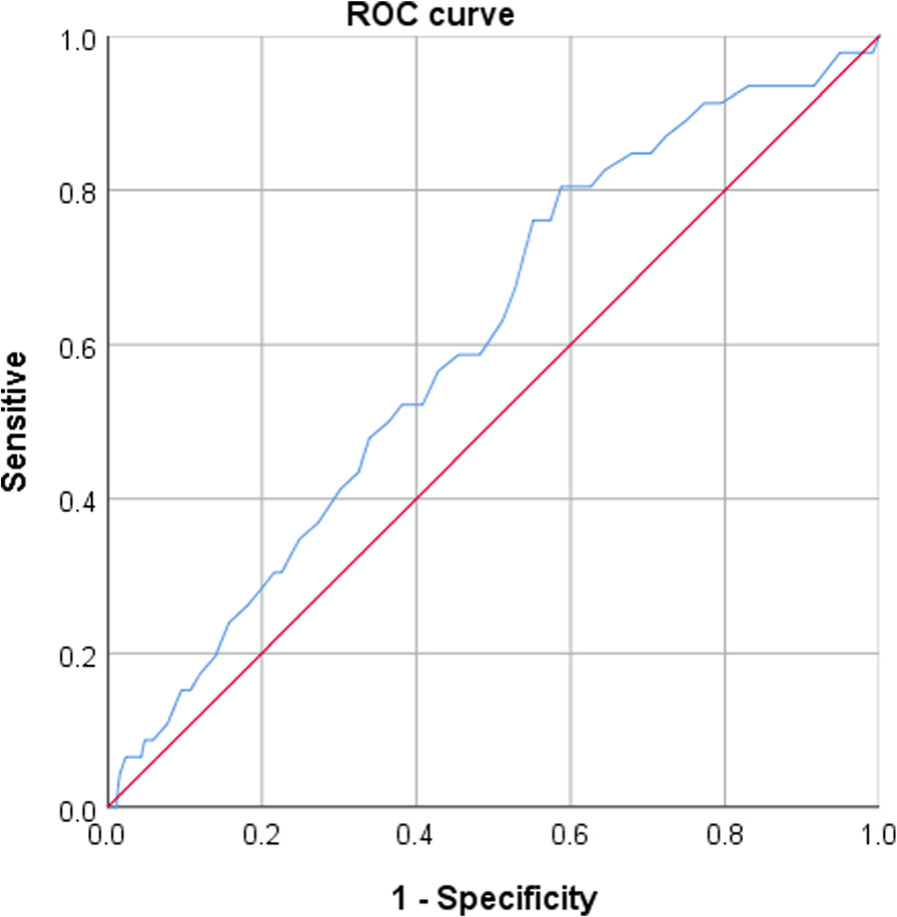
The inflection point of the ROC curve corresponded to the age above 41 years (*p* = 0.018; AUC, 0.603; 95%CI, 0.525 to 0.681; sensitivity = 0.804, specificity of 0.412)

The optimal cutoff value for D-dimer was identified at 1.75 mg/L, with a sensitivity of 0.860 and specificity of 0.174 (*p* = 0.021; AUC, 0.604; 95%CI, 0.517 to 0.692) (Fig. 1). The optimal cutoff value for age was 41 years, with a sensitivity of 0.804 and specificity of 0.412 (*p* = 0.018; AUC, 0.603; 95%CI, 0.525 to 0.681) (Fig. 2).

Table 1 presented the univariate analyses. DVT and non-DVT group differed in living area, anesthesia pattern, age in continuous or categorical variable, surgical duration, LDH, TP, HBDH, Na + and K+ concentration, HGB level, D-dimer (≥ 1.75 mg/L), and OSM.

**Table 1:** Univariate analyses of risk factors associated with DVT following surgeries of tibial plateau fracture

Variables	Number (%) of DVT (*n* = 46)	Number (%) of non-DVT (*n* = 941)	***P***
**Gender (male)**	30 (65.2)	573 (60.9)	0.557
**Age (years)**	49.8 ± 13.1	45.0 ± 13.9	0.020
**18–40**	9 (19.6)	388 (41.2)	0.003
**≥ 41**	37 (80.4)	553 (58.8)	
**Living area**			< 0.001
**Rural**	27(58.7)	577(61.2)	
**Urban**	19(39.1)	365(38.8)	
**BMI (kg/m**^**2**^**)**			0.934
**18.5–23.9**	14 (30.4)	306 (32.5)	
**< 18.5**	1 (2.2)	18 (1.9)	
**24–27.9**	20 (43.5)	389 (41.3)	
**≥ 28.0**	11 (23.9)	228 (24.3)	
**Diabetes mellitus**	5 (10.9)	126 (13.4)	0.623
**Hypertension**	7 (15.2)	160 (17.0)	0.752
**Cerebrovascular disease**	0	12(1.3)	0.441
**Chronic heart disease**	2 (4.3)	40 (4.3)	0.975
**Chronic liver disease**	2 (4.3)	45 (4.8)	0.893
**History of any surgery**	7 (15.2)	109 (11.6)	0.455
**Allergy to any medications**	5 (10.9)	116 (12.3)	0.768
**Current smoking**	6 (13.0)	123 (13.1)	0.996
**Alcohol consumption**	8 (17.4)	83 (8.8)	0.050
**Preoperative duration (day)**	7.1 ± 7.8	6.8 ± 6.7	0.781
**Total hospital stay**	29.8 ± 26.8	18.6 ± 22.2	0.001
**Mechanism (high-energy)**	30(65.2)	608(64.6)	0.933
**Open fracture**	5(10.9)	44(4.7)	0.059
**ASA class**			0.085
I	4 (8.7)	134 (14.2)	
II	31(67.4)	684 (72.7)	
III or above	11 (23.9)	123 (13.1)	
**Fracture type (Schartzker)**			0.397
**I–IV**	31 (67.4)	669 (71.1)	
**V–VI**	15 (32.6)	272 (28.9)	
**Anesthesia (general)**	28(60.9)	369(39.2)	0.003
**Open reduction (vs closed)**	34 (93.5)	96(91.2)	0.589
**Bone grafting (yes)**	13(28.3)	186(19.8)	0.161
**Surgical duration**	182.4 ± 77.0	148.6 ± 82.0	0.012
**Perioperative blood transfusion**	7 (15.2)	97 (10.3)	0.290
**Operation timing**			0.942
**Day**	45(97.8)	922(98.0)	
**Night**	1(2.2)	19(2.0)	
**TP (< 60 g/L)**	19 (41.3)	242 (25.7)	0.019
**ALB (< 35 g/L)**	14 (30.4)	189 (20.1)	0.090
**A/G**			0.111
**1.2–2.4**	38 (82.6)	835 (89.8)	
**< 1.2**	7(15.2)	66(7.0)	
**> 2.4**	1(2.2)	30(3.2)	
**ALT (> 40 U/L)**	7(15.2)	173(18.4)	0.587
**AST (> 35 U/L)**	6(13.0)	128(13.6)	0.914
**TBIL (> 21 umol/L)**	4(8.7)	78(8.3)	0.922
**DBIL (> 6 umol/L)**	17(37.0)	257(27.3)	0.154
**IBIL (> 14 umol/L)**	8(17.4)	142(15.1)	0.671
**ALP (> 100)**	3(6.5)	38(4.0)	0.410
**HCRP (> 8 mg/L)**	37(80.4)	627(66.6)	0.051
**LDH (> 250 U/L)**	16(34.8)	198(21.0)	0.027
**HBDH (> 182 U/L)**	13(28.3)	153(16.3)	0.034
**TC (> 5.2 mmol/L)**	3(6.5)	118(12.5)	0.224
**TG (> 1.7 mmol/L)**	4(8.7)	163(17.3)	0.128
**HDL-C (< 1.1 mmol/L)**	23(50.0)	365(38.8)	0.128
**LDL-C (> 3.37 mmol/L)**	6(13.0)	129(13.7)	0.898
**VLDL (> 0.78 mmol/L)**	4(8.7)	158(16.8)	0.148
**Na + (< 135 mmol/L)**	23(50.0)	263(27.9)	0.001
**K+ (mmol/L)**			0.001
**3.5–5.5**	40(87.0)	874(92.9)	
**< 3.5**	3(6.5)	60(6.4)	
**> 5.5**	3(6.5)	7(0.7)	
**UA (> upper limit)**	1(2.2)	77(8.2)	0.140
**WBC (> 10 × 10**^**9**^**/L)**	20(43.5)	290(30.8)	0.071
**LYM (< 1.1 × 10**^**9**^**/L)**	17(37.0)	266(28.3)	0.203
**MON (> 0.6 × 10**^**9**^**/L)**	30(65.2)	562(59.7)	0.458
**RBC < lower limit**	21(45.7)	367(39.0)	0.367
**HGB < lower limit**	31(67.4)	495(52.6)	0.049
**HCT < lower limit**	34(73.9)	571(60.7)	0.072
**MCV (fL)**			0.830
**82–100**	43 (93.5)	875 (93.0)	
**< 82**	1 (2.2)	34 (3.6)	
**> 100**	2 (4.3)	32 (3.4)	
**MCH (pg)**			0.585
**27–34**	42 (91.3)	861 (91.5)	
**< 27**	3 (6.5)	39 (4.1)	
**> 34**	1 (2.2)	41 (4.4)	
**MCHC (< 316 g/L)**	3(6.5)	26(2.8)	0.134
**PDW (%)**			0.197
**12–18.1**	42 (91.3)	829 (88.1)	
**< 12**	2 (4.3)	96 (10.2)	
**> 18.1**	2 (4.3)	16 (1.7)	
**D-dimer (> 0.50 mg/L)**	32 (69.6)	528 (56.1)	0.072
**D-dimer (> 1.76 mg/L)**	22 (47.8)	231 (24.5)	< 0.001
**OSM < 260 mOsm/L**	10 (21.7)	85 (9.0)	0.004
**PLT > 300 × 10**^**9**^**/L**	15 (32.6)	200 (21.3)	0.068
**NEUT > 6.3 × 10**^**9**^**/L**	27 (58.7)	441 (46.9)	0.117
**FBG** (> 6.1 mmol/L)	16 (34.8)	318 (33.8)	0.890
**RDW (> 16.5%)**	5 (5.1)	54 (3.8)	0.506

*BMI* body mass index; *ASA* American Society of Anesthesiologists; *RBC* red blood cell, reference range: females, 3.5–5.0 **×** 10^12^/L; males, 4.0–5.5 **×** 10^12^/L. *HGB* hemoglobin, reference range: females, 110–150 g/L; males, 120–160 g/L; *FBG* fasting blood glucose; *HCT* hematocrit, 40–50%; *WBC* white blood cell; *NEUT* neutrophile; *LYM* lymphocyte; *PLT* platelet, 100–300 **×** 10^9^/L; *TP* total protein; *ALB* albumin; *RDW* red cell distribution width; *PDW* platelet distribution width; *TC* total cholesterol; *TG* triglyceride; *LDL-C* low-density lipoprotein; *HDL-C* high-density lipoprotein; *VLDL* very low-density lipoprotein

In the multivariate model, the abovementioned 13 variables, together with open fracture (*p* = 0.059), alcohol consumption (*p* = 0.050), ASA (*p* = 0.085), ALB (*p* = 0.090), HCRP (*p* = 0.051), WBC (*p* = 0.071), HCT (*p* = 0.072), D-dimer > 0.5 mg/L (*p* = 0.072), and PLT (*p* = 0.068) were included. At the final model, five risk factors were identified as independent factors associated with DVT, including age (≥ 41 vs < 41 years), general anesthesia (vs regional), hyponatremia, prolonged surgical duration, and D-dimer ≥ 1.75 mg/L (Table 2). The H-L test demonstrated the good fitness of the final model (*X*^2^ = 10.406, *p* = 0.109; Nagelkerke *R*^2^ = 0.068).

**Table 2:** Multivariate analysis of factors associated with DVT following surgeries of tibial plateau fractures

Variables	OR	95%CI (lower limit)	95%CI (upper limit)	***P***
Age (≥ 41 vs < 41 years)	3.08	1.43	6.61	0.004
Anesthesia (general vs regional)	2.08	1.12	3.85	0.021
Hyponatremia (< 135 mmol/L)	2.21	1.21	4.06	0.010
Surgical time (increase of every 15 min)	1.04	1.01	1.07	0.017
D-dimer (≥ 1.75 mg/L)	2.79	1.34	4.83	0.004

## Discussion

In the present study, we used a large-sample prospective cohort to address the epidemiologic characteristics of DVT following surgeries of tibial plateau fractures. We found the overall incidence of DVT following tibial plateau fracture was 4.7%, with 1.0% for proximal and 3.7% for distal DVT. Patients with a postoperative DVT were associated with a significantly prolonged total hospitalization stay by 11.2 days. Five risk factors were identified to be independently associated with postoperative DVT, including age (≥ 41 vs < 41 years), general anesthesia, hyponatremia, prolonged surgical time, and elevated D-dimer level (≥ 1.75 mg/L).

Several studies have reported the DVT following surgeries of lower extremity fractures, with greatly variable incidence rates [10, 14–17], but few studies were specified at tibial plateau fractures, especially the postoperative DVT. In a cohort of specific isolated lower extremity fractures, Wang et al. [16] reported the incidence rate of postoperative DVT was 45.4% in 176 tibial plateau fractures, and all the patients received thromboprophylaxis consisting of low molecular weight heparin (LMWH) and pneumatic compression with foot pump before and after surgery. Goel et al. [18] conducted a prospective randomized trial to compare the incidence of DVT following fractures below the knee and found the lower rate of DVT in group of patients receiving LMWH than that of those receiving placebo (8.7% vs 12.6%). In this study, we found a lower rate of DVT, that was 4.7%, relatively lower than the previous reports. The following may be used to account for such difference. Firstly, intermuscular vein thrombosis was excluded in this study due to its less clinical significance, whereas the studies both by Wang et al. [16] and Goel et al. did not distinguish among them. Secondly, we only investigated the in-hospital rate of DVT, so the follow-up was relatively shorter, and our data also showed that 27% of DVTs were detected 2 weeks after surgery. In the study by Goel et al. [18], the follow-up was lasted until 12 weeks, 7-fold as long as ours (average, 12.6 days). Thirdly, patients with preoperative DVTs were also excluded, and for these patients, their risk of postoperative DVT demonstrated to be higher than those without a preoperative DVT [19]. Fourthly, patients in this study were younger (mean, 45.6 years) than that (mean, 62.8 years) of Wang et al.’s study [10], and age has always been a risk factor for DVT [17]. Additionally, the ethnicity or race should also be the contributors, and in Asian population, the risk of DVT was lower [20, 21].

Proximal DVT, a well-known increased risk for pulmonary embolism, generally necessitate further management. In this study, it was found to be 1.0% following surgeries of tibial plateau. In comparison, Goel et al. [18] found no proximal DVT either in the experimental or placebo group; but Wang et al. [10] found a rate of 4.5% of proximal DVT following surgeries of tibial plateau fractures, and we inferred advanced age was still an important influential factor. Some authors suggested the risk of proximal DVT was related to the fracture sites that were higher in fractures more proximal to the hip [10, 22, 23]. Anyhow, we did not ignore the role of distal DV, and in some study, it demonstrated to present a similar risk of pulmonary embolism as proximal DVT [24]. We also observed the relatively few incident DVTs in the bilateral (rate, 5.1%) or even only in uninjured extremity (rate, 2.0%), which was consistent with the previous finding [18, 22]. Obviously, bilateral examination was worthwhile for potential DVTs in the uninjured extremity.

Age, either in continuous form or in categorical form, was identified to be independently associated with DVT in studies [18, 25] or was listed an important factor in guideline [26]. Of interest was the cutoff value of age set as 40 years more or less in these studies, consistent with our finding. An important reason might be the fact that patients at age of 40–44 years were more likely the sustainers of high-energy trauma and the resultant complex fractures, e.g., Schatzker V–VI fractures [27], which was also associated with DVT [18]. We used ROC curve to determine the optimal cutoff value of age (41 years) with a relatively high sensitivity of 0.804, suggesting the applicability of age in initial screening for DVT.

Although previous studies demonstrated that delay to surgical intervention was associated with risk of DVT [10, 28], we were unable to identify the association in this study. According to Goel et al. [18], the reduced time of immobilization before surgery reduce the risk of DVT, and in their study, they found a rate of 8.7% of DVT following surgery of lower extremity fracture within 48 h of fracture; however, this contrasted with another finding of 3.9% rate of DVT in patients treated with plaster immobilization who received thromboprophylaxis [29]. In a previous study, Wilson et al. [30] suggested that the blood coagulation was highest in 3–7 days after fracture, which could partly explain why more authors tend to operate within 48 h after fracture for reduction of DVT.

D-dimer level in plasma reflects the secondary increased fibrinolytic activity and the hypercoagulability, which is a well-established sensitive marker of thrombotic events. In clinical practice, 0.5 mg/L was generally used as a cutoff value, but it could not exhibit a satisfactory differentiation in diagnosis of DVT in this study. Given its clinical importance, we used ROC to determine that the optimal cutoff value of D-dimer was 1.75 mg/L, and above which was identified to be independently associated with DVT. The high sensitivity of 0.860 showed its usefulness in screening for suspected thrombotic events, and this figure was in range of the reports, from 76 to 93% based on different settings [31–33]. But the low specificity remained an issue, being only 0.174 in this study. Similar as ours, Zhang et al. [34] also re-defined the cutoff of D-dimer level as preoperative 4.01 mg/L and postoperative 5.03 mg/L in traumatic fractures, and they found the lower sensitivity (preoperative, 0.713; postoperative, 0.639) and higher specificity (preoperative, 0.448; postoperative, 0.705) in diagnosis of perioperative DVT. It is pity that we were unable to obtain data on postoperative D-dimer level, and their role required to be determined in the future.

In addition, we also identified the hyponatremia, general anesthesia, and prolonged surgical duration were independently associated with the formation of postoperative DVT. Hyponatremia is common in trauma events and was found to involve 29.0% of our patients. It is reported that both hyper- or hyponatremia were associated with the increased risk of venous thromboembolism or even mortality [35]. Therefore, correction of preoperative saline imbalance is of importance in reduction or prevention of postoperative DVT. Prolonged surgical duration by 15 min was identified to be associated with 4% increased risk of DVT, consistent with the Abelseth et al.’s [22] finding that prolonged time ≥ 105 min was associated with thromboembolic disease in lower extremity fracture distal to the hip. But this factor should be regarded dialectically. In more cases, prolonged surgical duration was a marker of difficulty in management of more complex fracture, soft tissue injury or inexperience of surgeons. On the other hand, some external factors such as operation night, surgeon fatigue, cooperation with assistants, and obese patient also affect the surgical process. General anesthesia as an important factor for DVT was extensively discussed in trauma [36] or arthroplasty [37, 38]; however, it was firstly reported as specified at tibial plateau fracture. The might be explained by the fact that regional anesthesia allowed early mobility and improved the postoperative functional outcome and pain relief after surgery [39].

Although most of these factors might not be easily modifiable, they did aid in counseling by patients about their risk of postoperative DVT. We suggest the use of regional anesthesia as much as possible when both local and general anesthesia were indicated. In addition, positive correction of preoperative imbalance of sodium ions and optimized surgical scheme, improved surgical skills, and cooperation with operation room staff to reduce surgical duration were feasible in prevention or reduction of postoperative DVT formation.

There were several limitations in this study. Firstly, as other multivariate analyses, we could not include all the potential factors that affect the occurrence of DVT, such as duration of immolization of the injured extremity before and after operation [11]. Secondly, for some uncommon medical conditions or comorbidities such as chronic nephrosis or long-term use of glucocorticoid, it is almost impossible to confirm their association with DVT. Thirdly, in this study, we used various types of LMWH, single dose (range, 2500–4100 IU), and usage days in variety, but we could not evaluate their respective effect in DVT formation. Fourth, we identified the association rather than the causation between variables and DVT; therefore, these results should be interpreted with caution.

## Conclusion

In summary, the incidence of DVT following surgeries of tibial plateau fracture was 4.7%, with 1.0% for proximal and 3.7% for distal DVT. Five risk factors were identified to be independently associated with postoperative DVT, including age (≥ 41 vs < 41 years), general anesthesia, hyponatremia, prolonged surgical time, and elevated D-dimer level (≥ 1.75 mg/L). Although most of them might not be easily modifiable, they were conducive to the individualized assessment, risk stratification, and development of targeted prevention programs.

## Data Availability

The data and materials contributing to this article may be made available upon request by sending an e-mail to the first author.

## Abbreviations

DVT: Deep venous thrombosis
DUS: Duplex ultrasonography
SSIOS: Surgical Site Infection in Orthopaedic Surgery
LMWH: Low molecular weight heparin
